# Chemical gas sensor array dataset

**DOI:** 10.1016/j.dib.2015.01.003

**Published:** 2015-02-16

**Authors:** Jordi Fonollosa, Irene Rodríguez-Luján, Ramón Huerta

**Affiliations:** BioCircuits Institute, University of California, San Diego, La Jolla, CA 92093, USA

**Keywords:** Chemometrics, Machine olfaction, Electronic nose, Chemical sensing, Machine learning

## Abstract

To address drift in chemical sensing, an extensive dataset was collected over a period of three years. An array of 16 metal-oxide gas sensors was exposed to six different volatile organic compounds at different concentration levels under tightly-controlled operating conditions. Moreover, the generated dataset is suitable to tackle a variety of challenges in chemical sensing such as sensor drift, sensor failure or system calibration. The data is related to “Chemical gas sensor drift compensation using classifier ensembles”, by Vergara et al. [1], and “On the calibration of sensor arrays for pattern recognition using the minimal number of experiments”, by Rodriguez-Lujan et al. [2]

The dataset can be accessed publicly at the UCI repository upon citation of: http://archive.ics.uci.edu/ml/datasets/Gas+Sensor+Array+Drift+Dataset+at+Different+Concentrations

**Specifications table**

**Table:** 

Subject area	*Chemistry*

More specific subject area	*Chemometrics, Machine Olfaction, Electronic Nose, Chemical Sensing, Machine Learning*
Type of data	*Text Files*
How data was acquired	*Metal Oxide (MOX) gas sensors provided by Figaro Inc. (TGS2600, TGS2602, TGS2610, TGS2620; four of each type) exposed to different gas conditions over a period of 36 months.*
Data format	*Processed*
Experimental factors	*For each measurement a 128-component vector is processed from the sensors׳ responses to extract steady-state and transient features.*
Experimental features	*Sensors were exposed to clean air before and after sample presentation.*
Data source location	*San Diego, California, US.*
Data accessibility	*Data in public repository:*
	*http://archive.ics.uci.edu/ml/datasets/Gas+Sensor+Array+Drift+Dataset+at+Different+Concentrations*
	*Citation of*[1,2]*is required.*

**Value of the data**

• Response of the same chemical sensor array measured consistently over a period of 36 months. Drift in sensors׳ sensitivity can be evaluated over time.
• Extensive dataset (13,910 measurements) generated from chemical sensors exposed to six different volatiles, each volatile presented at different concentration levels. The problem can be formulated either as a classification problem to determine which gas is present or as a regression task to determine the gas concentration levels.
• The dataset can be utilized to address sensor drift, sensor failure, system calibration, sensor poisoning, among other common challenges in chemical sensing [1–4].
• It can also be applied to concept drift, active learning, and pattern recognition in Machine Learning.
• Dataset suitable for the benchmark of different Machine Learning techniques designed for chemical sensing.

## 1 Experimental design, materials and methods

### 1.1 Experimental setup

The chemical detection platform included 16 commercially available metal-oxide gas sensors manufactured and commercialized by Figaro Inc. The sensor array had four types of sensors (four of each type) tagged as TGS2600, TGS2602, TGS2610, TGS2620. Hence, the detection platform generates a multivariate response upon exposure to different volatiles.

The operating temperature of the sensors is controlled by the voltage applied to the built-in sensors׳ heaters. The voltage on the heaters was kept constant at 5 V.

**Fig. 1:**
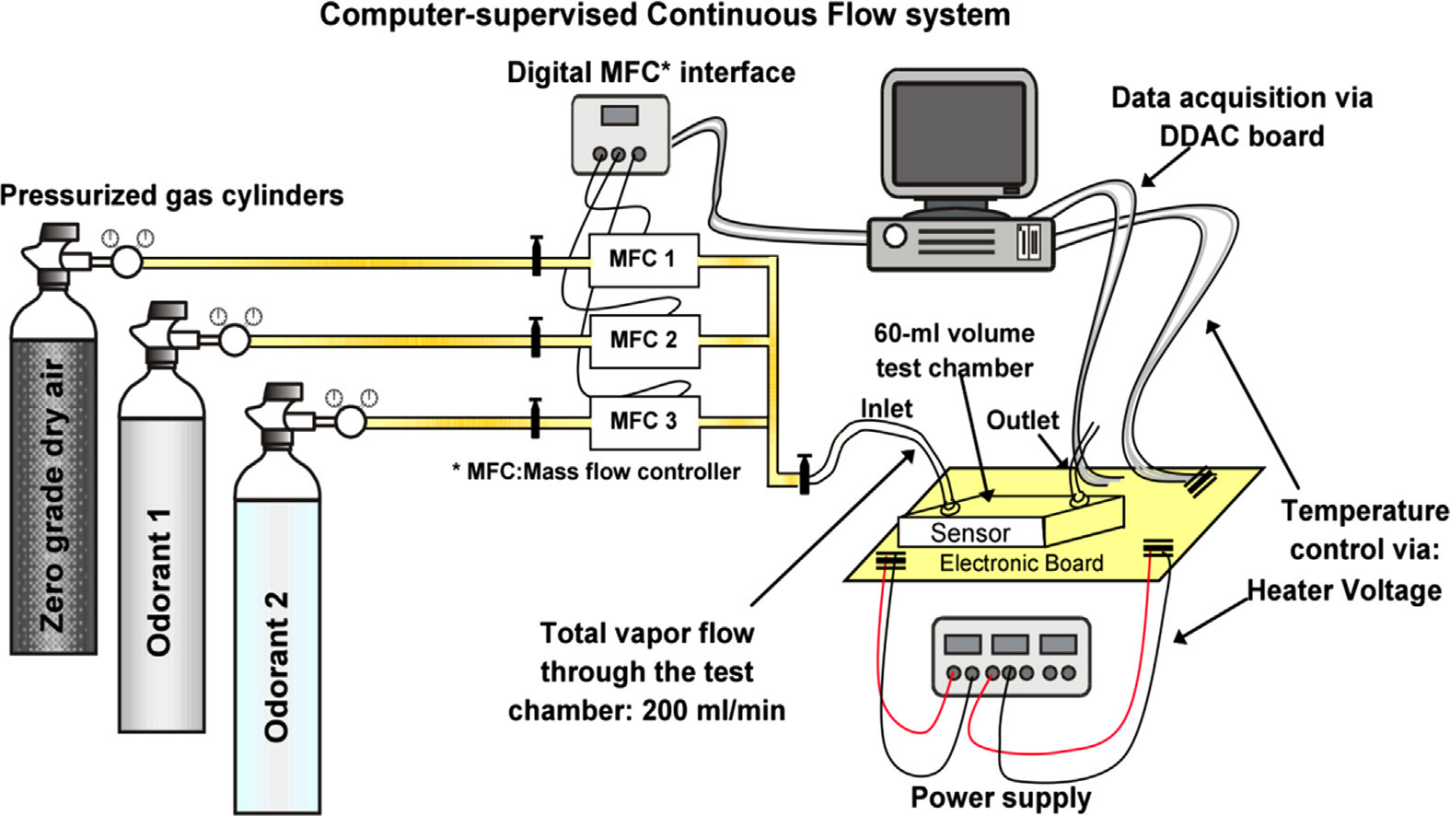
Experimental setup used for data acquisition. The sensor responses are recorded in the presence of the analyte in gaseous form diluted at different concentrations in dry air. The measurement system operates under a fully computerized environment with minimal human intervention, which provides versatility in conveying the chemicals of interest to the sensing chamber with high accuracy, and simultaneously to keep the total flow constant. Therefore, no changes in the flow or flow dynamics are reflected in the sensor response, (i.e., only the presence of a gas sample will induce the sensor conductivity to change). Moreover, since the system is continuously supplying gas to the sensing chamber (either clean dry air or a chemical component), the amount of gas molecules in the sensing chamber is homogeneously distributed.

We placed the sensor array into a 60 ml air-tight chamber where the volatiles of interest in gaseous form were injected in random order. The test chamber was attached in series to a vapor delivery system that provided the selected concentrations of the chemical substances by means of three digital mass flow controllers and calibrated gas cylinders. The total flow rate across the sensing chamber was set to 200 ml/min and kept constant for the whole measurement process. The entire measurement system setup was fully operated by a computerized environment and provided versatility for setting the concentrations with high accuracy and in a highly reproducible manner (see Fig. 1).

The dynamic response of each sensor was recorded at a sample rate of 100 Hz. Hence, each measurement produced a 16-channel time series sequence. The channels were paired with the sensors to acquire sensors׳ responses. Each pair remained unaltered for the whole dataset acquisition. The order of the sensors in the dataset is as follows (CH0-CH15): TGS2602; TGS2602; TGS2600; TGS2600; TGS2610; TGS2610; TGS2620; TGS2620; TGS2602; TGS2602; TGS2600; TGS2600; TGS2610; TGS2610; TGS2620; TGS2620.

## 2 Methods

To generate the dataset, we adopted a measurement procedure consisting of the following three steps. First, in order to stabilize the sensors and measure the baseline of the sensor response, we circulated synthetic dry air (10% R.H.) through the sensing chamber during 50 s. Second, we randomly added one of the analytes of interest to the carrier gas and made it circulate through the sensor chamber during 100 s. Finally, we re-circulated clean dry air for the subsequent 200 s to acquire the sensors׳ recovery and have the system ready for a new measurement.

The sensor array was exposed to six different volatiles, each of them at different concentration levels (see Table 1). Table 2 shows the data distribution over the 36-month period. For processing purposes, the dataset is organized into ten batches, each containing the number of measurements per class and month indicated in Table 2. This reorganization of data was done to ensure having a sufficient number of experiments in each batch, as uniformly distributed as possible. Note that a few measurements, mainly in batch 7, appear at lower concentration levels than detailed in Table 2. This concentration mismatch is due to some experimental error. For the sake of completeness, we decided to include those samples in the dataset.

### 2.1 Feature extraction

MOX gas sensors typically describe a monotonically smooth change in the conductance of the sensing layer due to the adsorption/desorption reaction processes of the exposed chemical analyte substance.

We represented each time series with an aggregate of eight features reflecting the sensor response. In particular, we considered two distinct types of features in the creation of this dataset: two steady-state features and six features reflecting the sensor dynamics.

The steady-state features include the amplitude of the resistance change, and its normalized value. The transient features were extracted based on the exponential moving average (EMA) to reflect the sensor dynamics of the increasing/decaying transient portion of the sensor responses [5]. The EMA transform evaluates the rising/decaying portions of the sensor resistance by considering the maximum/minimum values of y[k] of the following first-order digital filter:

y[k]=(1−α)y[k−1]+α(x[k]−x[k−1])

where 0<*α*<1 is the smoothing parameter of the filter and *x*[*k*] is the acquired value at time *k*. Since different values of *α* provide different feature values and different information of the transient response, we computed the EMA filter for three values of *α*=0.1, 0.01, 0.001 for both the rising and the decaying stages. Therefore, each of the 16 sensors used in the study contributes with 8 features, thereby yielding a 128-element feature vector per measurement.

## Figures and Tables

**Table 1:** Tested volatiles and concentration levels.

Volatile	Tested concentration levels
Ammonia	50, 60, 70, 75, 80, 90, 100, 110, 120, 125, 130, 140, 150, 160, 170, 175, 180, 190, 200, 210, 220, 225, 230, 240, 250, 260, 270, 275, 280, 290, 300, 350, 400, 450, 500, 600, 700, 750, 800, 900, 950, 1000
Acetaldehyde	5, 10, 13, 20, 25, 30, 35, 40, 45, 50, 60, 70, 75, 80, 90, 100, 120, 125, 130, 140, 150, 160, 170, 175, 180, 190, 200, 210, 220, 225, 230, 240, 250, 275, 300, 500
Acetone	12, 25, 38, 50, 60, 62, 70, 75, 80, 88, 90, 100, 110, 120, 125, 130, 140, 150, 170, 175, 180, 190, 200, 210, 220, 225, 230, 240, 250, 260, 270, 275, 280, 290, 300, 350, 400, 450, 500, 1000
Ethylene	10, 20, 25, 30, 35, 40, 50, 60, 70, 75, 90, 100, 110, 120, 125, 130, 140, 150, 160, 170, 175, 180, 190, 200, 210, 220, 225, 230, 240, 250, 275, 300
Ethanol	10, 20, 25, 30, 40, 50, 60, 70, 75, 80, 90, 100, 110, 120, 125, 130, 140, 150, 160, 170, 175, 180, 190, 200, 210, 220, 225, 230, 240, 250, 275, 500, 600
Toluene	10, 15, 20, 25, 30, 35, 40, 45, 50, 55, 60, 65,70, 75, 80, 85, 90, 95, 100

**Table 2:** Data distribution over the 36 months.

Batch	Months	Number of samples					
		Ethanol	Ethylene	Ammonia	Acetaldehyde	Acetone	Toluene
1	1,2	83	30	70	98	90	74
2	3, 4, 8, 9, 10	100	109	532	334	164	5
3	11, 12, 13	216	240	275	490	365	0
4	14, 15	12	30	12	43	64	0
5	16	20	46	63	40	28	0
6	17, 18, 19, 20	110	29	606	574	514	467
7	21	360	744	630	662	649	568
8	22, 23	40	33	143	30	30	18
9	24, 30	100	75	78	55	61	101
10	36	600	600	600	600	600	600
